# Evidence based decision making between PCI and CABG

**DOI:** 10.5935/abc.20190076

**Published:** 2019-05

**Authors:** Carlos Collet

**Affiliations:** Cardiovascular Center OLV, Aalst - Belgium

**Keywords:** Myocardial Revascularization/mortality, Percutaneous Coronary Intervention, Drug-Eluting Stents, Stents, Coronary Vessels, Randomized Controlled Trials, Clinical Decision Making

For the last five decades, coronary artery bypass grafting (CABG) surgery has been recommended for patients with unprotected left main (ULM) and multivessel coronary artery disease (MVD).^1^ In these populations, CABG reduces mortality compared to medical management.^2^ In patients with MVD, several randomized clinical trials established the superiority of CABG over percutaneous coronary interventions (PCI) in terms of hard clinical endpoints.^3,4^ In the ULM subgroup of the SYNTAX I trial, comparable outcomes between PCI and CABG were observed at five years.^5^ This finding triggered the design and execution of the EXCEL and NOBLE trials that confirmed equipoise in major adverse cardiovascular and cerebral events between PCI and CABG in patients with ULM coronary artery disease (CAD).^6,7^

The accumulation of evidence has allowed to better understand which patients may benefit from a determined revascularization strategy.^8^ In the current issue of the Journal, Negreiros de Andrade et al.^9^ present a study-level meta-analysis comparing clinical outcomes after PCI and CABG in patients with ULMCAD and MVD. The authors should be commended for the stratified analysis aiming at providing practical information for the cardiovascular community. Based on the current state of evidence we can state that 1) in patients with ULMCAD, PCI can be considered an alternative to CABG in patients with low anatomical complexity, and 2) patients with MVD have better clinical outcomes when treated with CABG. When these two populations were combined, the present meta-analysis showed an early (<30 days) benefit of PCI in terms of mortality and stroke, and long-term advantage of CABG in death and myocardial infarction.

Heart team's interaction is the mainstay of the clinical decision-making process. Key clinical factors such as age, sex, the presence of diabetes mellitus, chronic obstructive pulmonary disease (COPD) and left ventricular ejection fraction should be accounted for in the selection between PCI and CABG. In addition, anatomical consideration based on the presence of isolated ULMCAD and/or MVD must also influence the treatment decision.^8^ The SYNTAX score II was developed to aid the heart team in the decision-making process considering the interaction of between clinical variables and anatomical complexity. The score incorporates the clinical variables with the anatomical SYNTAX score providing a treatment recommendation (i.e. PCI or CABG) based on predicted 4-year mortality.^10^ Mortality estimation based on individual patient profiles enhances heart team discussion, patient's information and shared decision making. Furthermore, the SYNTAX II score has been validated in contemporary clinical trials; in the EXCEL trial patients randomized to PCI in whom the SYNTAX score II recommended CABG had higher all-cause mortality at 3-year follow-up.^11^ Moreover, in the SYNTAX II study, patients with MVD selected based on a mortality risk equipoise between PCI and CABG had similar outcomes compared to a matched population undergoing CABG.^12,13^ A practical recommendation, supported by the findings of this meta-analysis are that: females, young patients, diabetics, low-ejection fraction and MVD with high anatomical complexity (e.g. high anatomical SYNTAX score) have better prognosis when treated with CABG, whereas in old patients, with COPD or ULMCAD with low anatomical complexity PCI is an acceptable alternative. Long term data (i.e. 10 years) from the original SYNTAX and FREEDOM have become available and showed a persistent advantage of CABG over PCI in patients with MVD.^3^ Long term clinical follow-up of patients with ULMCAD included in EXCEL and NOBLE are awaited to further define the best treatment strategy.

Further refinement in the evaluation of patients with ULM and MVD can be achieved using coronary physiology indexes. Systematic use of fractional flow reserve has been shown to reduce the number of lesions that appear to be angiographically significant, reclassify a significant proportion of patients to lower SYNTAX score tertiles and improve clinical outcomes compared to angiographic-guided PCI and optimal medical therapy.^14-16^ A Comparison of Fractional Flow Reserve-Guided Percutaneous Coronary Intervention and Coronary Artery Bypass Graft Surgery in Patients With Multivessel Coronary Artery Disease (FAME 3) will further provide answers on the best revascularization strategy tailoring treatment decision based on coronary physiology. In the near future, virtual tool predicting functional improvement after PCI or CABG will further refine patients' selection potentially improving clinical outcomes in stable CAD.
